# 2-{*N*-[ω-(1-Benzylpiperidin-4-yl)alkyl]amino}-6-[(prop-2-yn-1-yl)amino]pyridine-3,5-dicarbonitriles Showing High Affinity for σ_1/2_ Receptors

**DOI:** 10.3390/ijms26031266

**Published:** 2025-01-31

**Authors:** Winnie Deuther-Conrad, Dirk Schepmann, Isabel Iriepa, Francisco López-Muñoz, Mourad Chioua, Bernhard Wünsch, Abdelouahid Samadi, José Marco-Contelles

**Affiliations:** 1 Helmholtz-Zentrum Dresden-Rossendorf (HZDR), Department of Neuroradiopharmaceuticals, Institute of Radiopharmaceutical Cancer Research, D-04318 Leipzig, Germany; w.deuther-conrad@hzdr.de; 2 Institut für Pharmazeutische und Medizinische Chemie, Universität Münster, Corrensstraße 48, D-48149 Münster, Germany; dirk.schepmann@uni-muenster.de (D.S.); wuensch@uni-muenster.de (B.W.); 3 Departamento de Química Orgánica y Química Inorgánica, Instituto de Investigación Química “Andrés M. del Río” (IQAR), Universidad de Alcalá, Alcalá de Henares, 28805 Madrid, Spain; isabel.iriepa@uah.es; 4 Grupo DISCOBAC, Instituto de Investigación Sanitaria de Castilla-La Mancha (IDISCAM), 28805 Madrid, Spain; 5 Faculty of Health Sciences–HM Hospitals, University Camilo José Cela, 28692 Madrid, Spain; flopez@ucjc.edu; 6 HM Hospitals Health Research Institute, 28015 Madrid, Spain; 7 Neuropsychopharmacology Unit, “Hospital 12 de Octubre” Research Institute, 28041 Madrid, Spain; 8 Institute of General Organic Chemistry (CSIC), C/Juan de la Cierva 3, 28006 Madrid, Spain; m.chioua@csic.es; 9 Department of Chemistry, College of Science, United Arab Emirates University, Al Ain 15551, United Arab Emirates; 10 Centre for Biomedical Network Research on Rare Diseases (CIBERER), CIBER, ISCIII, 46010 Madrid, Spain

**Keywords:** ADME, computational chemistry, docking, multifunctional pyridines, sigma receptors

## Abstract

Sigma receptors (σRs) represent very attractive biological targets for the development of potential agents for the treatment of several neurological disorders. In the search for new small molecule drugs against neuropathic pain, we identified 2-{[2-(1-benzylpiperidin-4-yl)ethyl]amino}-6-[methyl(prop-2-yn-1-yl)amino]pyridine-3,5-dicarbonitrile (**5**) as a polyfunctionalized small pyridine with potent dual-target activities against acetylcholinesterase (AChE) (IC_50_ = 13 nM) and butyrylcholinesterase (BuChE) (IC_50_ = 3.1 µM), exhibiting high σ_1_R affinity (*K*_i_(hσ_1_R) = 1.45 nM) and 290-fold selectivity over the σ_2_R subtype. These results are in good agreement with those found in the molecular modeling of compound **5**. This is possibly due to the preferred combination in this molecule of a linker n = 2 connecting the *N*-Bn-piperidine motif to the C2 pyridine, without a phenyl group at C4, and a *N*-Me-substituted propargyl amine in the chain located at C6.

## 1. Introduction

Sigma receptors (σRs) are a type of singular receptor implemented in diverse biological facts and events. Two σRs subtypes are known: the σ_1_ receptor (σ_1_R) and σ_2_ receptor (σ_2_R). σRs have been involved in diverse human conditions, such as Alzheimer’s disease (AD), neuropathic pain, and cancer [1,2]. In spite of the fact that from a structural point of view σ_1_R and σ_2_R are distinct proteins, it has been possible to identify different ligands showing affinity for σ_1_R and/or σ_2_R [3], conceive new compounds that modulate these proteins, [4] and develop molecules for PET techniques [5].

σ_1_Rs play a key role in Ca^2+^ and other ion channel signaling processes in the regulation of the endoplasmic reticulum, as well as in mitochondrial activity [6]. σ_1_Rs are also involved in cognition and pain [7] and are affected by trophic factors [1,6]. Finally, it is well known that σ_1_R agonists have proved successful in the recovery of cognitive impairment in suitable animal models, mostly due to enhanced activity in the cholinergic and glutamatergic systems [8]. For appropriate models of pain, σ_1_R antagonists cancel sensory hypersensitivity, with σ_1_R agonists showing the reverse effect of σ_1_R antagonists [9,10]. These results have been applied with success to design new σ_1_R modulators [11,12]. The reduction in the central sensitization due to σ_1_R antagonists is of paramount importance for the treatment of pain in humans, particularly neuropathic pain [13,14], and for advanced clinical studies [15,16].

Regarding the structure–activity connections, some general trends have been observed [1]. Thus, ligands bearing a basic amino group in piperidines [17], such as haloperidol and (+)-MR200, [18] in ethylenediamines, [19,20] pyrimidines, [21] or in polyfunctionalized 1,3-dioxanes [22] and isoxazoles [23] are typical σ_1_R antagonists, although some exceptions have been reported [24]. Of particular interest, the σ_1_R antagonist S1RA [25,26] is a new chemical entity in phase II clinical tests for the therapy of neuropathic pain [27]. In this context, ligands bearing the *pyridine* motif have been barely analyzed as σ_1_/σ_2_R activators [28], and only two reports [29,30] have been communicated.

In our current research project, which aims to identify multitarget directed ligands showing σ_1_/σ_2_R affinity for AD and neuropathic pain [31], we have now considered polyfunctional pyridines **1**–**12** of type **I** (Table 1). Compounds of type **I** (Table 1) are polyfunctionalized pyridines substituted with cyano groups at 3 and 5 positions and bearing a proton (**Ia**,**b**) or a phenyl moiety (**Ic**,**d**) at 4 positions. In addition, compounds of type **I** (Table 1) are substituted at 2 positions with a 1-benzylpiperidin-4-yl moiety linked to the pyridine ring by a spacer (n = 0–4), which is responsible for the observed cholinesterase (ChE) inhibition properties of these ligands [32,33]. Furthermore, compounds of type **I** (Table 1) are substituted at 6 positions with a *N*-(prop-2-yn-1-yl)amino (**Ia**,**c**) or a *N*-methyl-*N*-(prop-2-yn-1-yl)amino (**Ib**,**d**) moiety, which is responsible for the presumed and observed neuroprotection and monoamine oxidase (MAO) A/B inhibition properties of these molecules [32,33]. As shown in Table S1 (Supporting Information), compounds **4**, **7**, and **10** were able to selectively and significantly inhibit AChE in the 1.1–1.7 nM range vs. BuChE, in the 530−840 nM range and among all the ligands, only compound **7** showed the capacity to inhibit MAO-A selectively (3950 nM).

Thus, we were curious to analyze whether ligands of type **I** (Table 1) incorporating *N*-benzyl-substituted piperidines linked to polyfunctionalized pyridines are able to modulate functional and biological responses in σ_1/2_R. It is important to highlight that, in agreement with all the pharmacophore models, which have the presence of one positively charged group with hydrophobic features in common, and almost all include a polar group [34,35], pyridines of type **I** (Table 1) possess a basic amino moiety as a positively ionizable group flanked by two hydrophobic regions. Consequently, in this work, we investigated the modulation of σ_1/2_Rs by pyridines **1**–**12** (Table 1).

**Table 1:** Binding affinities of pyridines **1**–**12** and reference compounds vs. σ_1_R and σ_2_R [36,37].

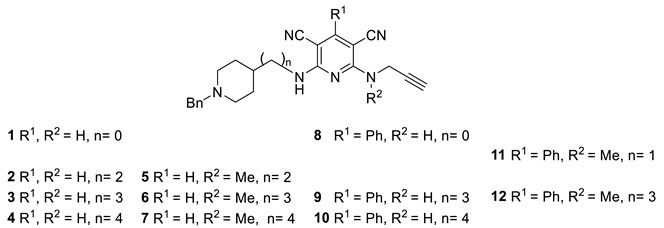			
Comp.	*K*_i_ Human σ_1_R ^a^ (nM)	*K*_i_ Rat σ_2_R ^b^ (nM)	Selectivity Index of Rat σ_2_R/Human σ_1_R
**1**	29.2 (23.9; 34.5)	173	5.9
**2**	7.57 ± 0.59	42 ± 8	5.6
**3**	2.97 ± 0.22	37 ± 5	13
**4**	3.97 ± 0.66	163 ± 50	41
**5**	**1.45 ± 0.43**	**389**	**270**
**6**	3.05 ± 1.27	129 ± 26	42
**7**	3.09 ± 1.25	288	93
**8**	27.3 (21.4; 33.2)	141	5.2
**9**	7.45 ± 4.32	104 ± 22	14
**10**	15.4 (12.3; 18.5)	39	2.5
**11**	**119 (157; 81.4)**	**55**	**0.46**
**12**	10.9 ± 1.9	393	36
**NE-100**	2.00 (1.9; 2.1)	n.d. ^c^	n.d. ^c^
**PB28**	1.9	n.d. ^c^	n.d. ^c^

^a^ *K*_i_ values for *h*σ_1_R were measured on membrane preparations of HEK-293 cells stably transfected with *h*σ1R using (+)-[^3^H]pentazocine as the radioligand. Non-specific binding of the radioligand was determined with 10 µM haloperidol. In each experiment, compounds were tested in technical triplicate in the range of 10^−11^–10^−5^ M. *K*_i_ values were calculated according to Cheng and Prusoff [36]. A single *K*_i_ value was obtained from a single experiment. The mean of the *K*_i_ values of two independent experiments is given, with the individual values of each experiment in parentheses. The mean of *K*_i_ values from ≥ three independent experiments is reported with the standard deviation (mean ± SD). ^b^ The σ_2_R affinity was determined using rat liver membrane preparations with the radioligand [^3^H]ditolylguani-dine in the presence of 100 nM (+)-pentazocine to mask σ_1_R binding sites. Non-specific binding was determined with 10 μM ditolylguanidine [37]. *K*_i_ values were calculated according to Cheng and Prusoff [36], and data from at least three independent experiments are represented, each performed in triplicate. The results are given as the mean (standard error of the mean (SEM)). ^c^ n.d. = not determined.

## 2. Results and Discussion

### 2.1. In Vitro Modulation of σ1R and σ2R by Pyridines of Type I

We investigated the interaction of pyridines **1**–**12** (Table 1) and reference standards **NE-100** and **PB28** with σ_1_R and σ_2_R (**Experimental Section 4.1.**), obtaining the results shown in Table 1 [36,37] using the established experimental protocols (see **Experimental**) [38].

Regarding the *K*_i_ values of the human σ_1_R (*h*σ_1_R), eleven out of the twelve compounds tested were bound towards *h*σ_1_R with high affinities, as reflected by *K*_i_ values below 30 nM (Table 1). However, the following relationships between the structural features of this particular family of compounds and the σ_1_R affinity can be identified as follows:

1. The linker between the 1-benzylpiperidine moiety and the pyridine ring plays a crucial role in σ_1_R affinity, as increasing the length from the amino group in compound **1** (n = 0, *K*_i_ = 29.2 nM) to an ethylamino (**2**: n= 2, *K*_i_ = 7.57 ± 0.59 nM), a propylamino (**3**: n = 3, *K*_i_ = 2.97 ± 0.22 nM) and to a butylamino group (**4**: n = 4, *K*_i_ = 3.97 ± 0.66 nM), resulted in increased *h*σ_1_R affinity.
2. The introduction of a methyl moiety at the *N*-(prop-2-yn-1-yl)amino substituent at C6 of ligands bearing R^1^ as H, such as **2**, **3**, and **4**, led to pyridines **5**, **6**, and **7**, significantly increasing their affinity from ligand **2** (*K*_i_ = 7.57 ± 0.59 nM) to pyridine **5** (*K*_i_ = 1.45 nM) with n = 2 as the linker, but had no remarkable effect on the transition from the ligands **3** and **4** to the pyridines **6** and **7** with n = 3 as the linker. However, the introduction of a methyl substituent for the R^2^ group in compound **9** (*K*_i_ = 7.45 nM) bearing R^1^ as Ph to yield compound **12** (*K*_i_ = 10.9 nM) significantly decreased the affinity.
3. The insertion of a phenyl group at C4 of the pyridine ring had no impact on the σ_1_R affinity for n = 0 as the linker, **1** vs. **8**, but significantly decreased the affinity from ligand **3** to compound **9** (n = 3), and from ligand **4** to compound **10** (n = 4). A similar decreasing σ_1_R affinity effect was induced by the insertion of a phenyl group at C4 on the *N*-methyl-propynylamine-substituted pyridine **6** (*K*_i_ = 3.05 nM) compared to compound **12** (*K*_i_ = 10.9 nM) for n = 3 as the linker.
4. Finally, compound **11** showed the lowest *h*σ_1_R affinity, most likely due to the combination of the sub-optimal distance of the two essential hydrophobic regions (n = 1) and the effects of the *N*-methyl-propynylamine and the phenyl-substituted pyridine moiety serving as the aromatic hydrophobic region.

As shown in Table 1, the ligand with the highest affinity for binding to *h*σ_1_R was 2-{[2-(1-benzylpiperidin-4-yl)ethyl]amino}-6-[methyl(prop-2-yn-1-yl)amino]pyridine-3,5-dicarbonitrile (**5**) (Table 1) with a *K*_i_ value of 1.45 ± 0.43 nM, which is in the range of the standard compounds **NE-100** (*K*_i_ = 2.00 nM) and **PB28** (*K*_i_ = 1.87 nM), followed by very potent ligands **3**, **6** and **7** with *K*_i_ values in the 3 nM range.

To determine the selectivity, the *K*_i_ values of rat σ_2_Rs (*r*σ_2_Rs) were determined, and the selectivity ratio (*K*_i_ rat σ_2_R/*K*_i_ *h*σ_1_R) was calculated. As shown in Table 1, ligands **5** and **7** are highly selective for *h*σ_1_R *vs r*σ_2_R, with compound **5** showing a selectivity of 270-fold, whereas compound **7** was only 93-fold more selective for hσ_1_R vs. *r*σ_2_R. In conclusion, ligand **5** showed high σ_1_R affinity and selectivity over the σ_2_R subtype. Very interestingly, among all the investigated compounds, only ligand 2-{[(1-benzylpiperidin-4-yl)methyl]amino}-6-[methyl(prop-2-yn-1-yl)amino]-4-phenylpyridine-3,5-dicarbonitrile (**11**) (Table 1) was 2-fold more affine for rat σ_2_R than for *h*σ_1_R (Table 1). This is possibly due to the combination of a linker n = 1 connecting the *N*-Bn-piperidine motif to the C2 pyridine in this molecule alone, with a phenyl group at C4, and a *N*-Me substituted propargyl amine in the chain located at C6.

Consequently, the docking analysis on compounds **3**, **5**, **6**, **9**, **11** and **12** (Table 1) was performed to determine and justify its binding affinity in silico.

### 2.2. Molecular Docking of Pyridines ***3***, ***5***, ***6***, ***9***, ***11*** and ***12*** with σ_1_R and σ_2_R

AutoDock Vina [39] and Discovery Studio were selected to perform the docking simulations and visualizations.

**Figure 1:**
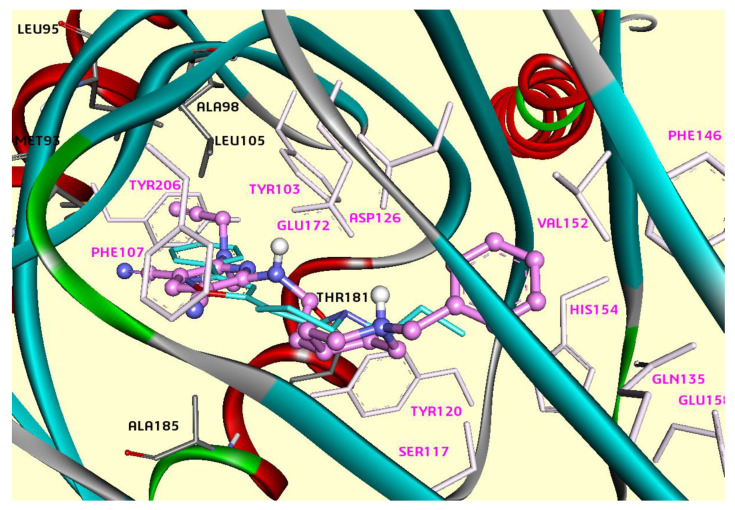
The modeled complex of the *h*σ_1_R with ligand **5** (pink). Binding energy: −11.2 kcal/mol. Flexible residues are shown by pink sticks. Compound PD144418 from the σ_1_R crystal structure (PDB: 5HK1) is shown by blue sticks.

We used a three-dimensional model of *h*σ_1_Rs [38] based on the crystal structure of the *h*σ_1_R model bound to the antagonist PD144418 (PDB ID: 5HK1). In our model, each ligand adapted to the receptor active site by rearranging the side chains of residues Tyr103, Glu172, Phe107, Asp126, Val152, Phe146, Gln135, His154, Glu158, Ser117, Tyr120, and Tyr206. The binding pocket comprised hydrophobic residues Val84, Trp89, Met93, Leu95, Leu105, Leu182, Phe107, Ile124, Trp164, and Tyr103, except for two acidic residues: Glu172 and Asp126. Glu172 and Tyr103 could be considered forms of the “binding dyad” that anchor the ligand to the receptor. The most active ligand, compound **5**, resembles the known binding pose of the antagonist PD144418, fully occupies the active site, and establishes several interactions with σ_1_R (Figure 1).

**Figure 2:**
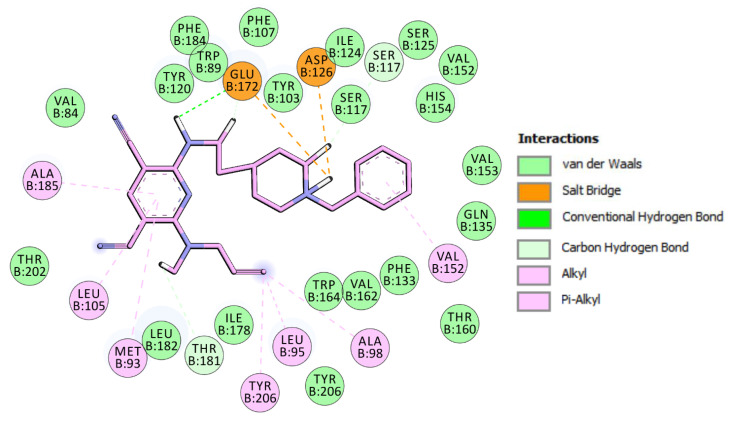
Two-dimensional representation of the interactions of ligand **5** (pink) in the active site of σ_1_Rs.

As shown in Figure 2, the propargylamine group is perfectly encased in the cavity lined by residues Tyr206, Ala98, Leu95, and Thr181. On the other hand, the pyridine ring establishes π-alkyl interactions with Leu105, Met93, and Ala185. Glu172 forms a hydrogen bond with the NH group, and carbon–hydrogen interactions are established with the CH_2_ group of the alkyl chain (Figure 2). The piperidinium moiety was found to involve salt bridge interactions with Asp126 and Glu172, as well as carbon–hydrogen interactions with Ser117. Finally, the phenyl ring interacted with Val152 via π–alkyl interactions.

The residue interactions of compounds **3**, **6**, **9** and **12** against human σ1Rs were also investigated to identify key residues crucial for ligand binding (Supporting Information).

The docking results show that compounds **9** and **12** occupy q similar binding site as compound **5** at σ1R [Figure 1 and Figure S1 (Supporting Information)] by displaying matching contacts between piperidinium nitrogen and Glu172 through a salt bridge. In addition, the hydrophobic contacts observed include those between the pyridine ring and Met93 (Figure S2, Supporting Information).

Meanwhile, prominent differences between the binding of compounds **3**, **6**, and compound **5** were also noted. Ligands **3** and **6** and compound **5** bind to this receptor in opposite directions. That is, compounds **3** and **6** bind to the receptor with the benzyl group proximal to the membrane, while compounds **5**, **9**, and **12** bind with the pyridine ring near the membrane (Figure S1, Supporting Information). Both compounds **3** and **6** show good docking scores (−12 kcal/mol and −11.7 kcal/mol, respectively).

Compounds **3** and **6** shared a common attractive charge and π–anion interactions with Glu172, as well as π–cation interactions with Tyr103 (Figure S2, Supporting Information).

The two most active ligands, **5** and **3**, also interacted directly through hydrogen bonds with Glu172 and Asp126, respectively, whereas compounds **6**, **9**, and **12** did not form any direct hydrogen bond with these key amino acids. These results show that NH is crucial to stabilizing the compounds in the active site. Compound **5**, with a linker of n = 2, interacts with the receptor through the NH group that connects the Bn–piperidine motif to the C2 position of the pyridine ring. In contrast, compound **3**, with a linker of n = 3, interacts via the NH group of the propargylamine moiety (Figure S2, Supporting Information). Additionally, compounds **9** (binding energy = −11.1 kcal/mol) and **12** (binding energy = −10.4 kcal/mol), bearing a phenyl group at the C4 position of the pyridine ring, showed a decrease in docking scores, and the Me-substitution on the propargylamine moiety had limited influence on their mechanism of interaction.

The molecular docking results showed that compounds **5**, **6**, **9**, **11**, and **12** adopted a similar orientation within the active site of the σ2R (Figure S3, Supporting Information). It was observed that the *N*-benzyl-piperidinium system of these compounds binds to the same pocket formed by His21, Met59, Phe66, Leu70, Leu111, Ileu114, Tyr147, Tyr150, Asp29, and Glu73. These two last key amino acids were involved in stabilizing the compounds in the active site. Glu73 was found to form π–anion interactions with the benzyl moiety, except for compound **5**. Asp29 interacts with the piperidinium moiety through salt bridges or attractive charge interactions (Figure S4, Supporting Information). Furthermore, for compounds **5**, **6**, **9**, and **12**, the alkyl-linker occupied the adjacent small site formed by Ile24, Ile28, Tyr50, and Asp56 (Figure S4, Supporting Information). In the case of compound **11**, which has the shortest alkyl linker, phenyl-substituted pyridine occupies the aforementioned site (Figure S4, Supporting Information). Finally, the substituted-pyridine moiety is located in the pocket formed by Leu46, Trp49, Phe54, and Val146.

Interestingly, compound **3**, the most active compound against σ2R, adopted an opposite orientation to that of the other compounds (Figure S3, Supporting Information) while still interacting with the same amino acids. In this situation, the substituted-pyridine moiety established π–anion interactions with Asp29, and the NH of the propargylamine group formed a hydrogen bond with Glu73. This network of key interactions may be responsible for the greater activity of compound **3** compared to the other ligands.

Although there is not much difference, the docking binding energy scores of the compounds **5** (−7.9 kcal/mol), **6** (−8.1 kcal/mol), **9** (−8.2 kcal/mol), **11** (−8.4 kcal/mol), and **12** (−7.8 kcal/mol) are consistent with the binding affinities against the σ2R.

### 2.3. Virtual ADME of Pyridines ***3***, ***5***, ***6***, ***9***, ***11***, and ***12***

The ADME (Absorption, Distribution, Metabolism, and Excretion) properties of compounds **3**, **5**, **6**, **9**, **11**, and **12** were theoretically calculated using the QikProp module of the Schrödinger suite (QikProp, Schrödinger, LLC, New York, NY, USA, 2024) in normal mode to evaluate their druggability. A wide range of physically significant descriptors and pharmacologically relevant properties were predicted and analyzed (Table S2, Supporting Information).

These findings indicate that compounds **3**, **5**, **6**, and **11** adhere to Lipinski’s rule of five [40]. Compounds **9** and **12** violate this rule once and twice, respectively. All compounds had most of the calculated descriptors and properties within the expected QikProp thresholds, except for the estimated number of hydrogen bonds that the solute could accept (accept HB). Aqueous solubility (QPlogS) is crucial for many ADME-related properties. Compounds **3**, **5** and **6** exhibited solubility values within the acceptable range (QPlogS = −5.437 to −6.156; limits −6.5 to 0.5; S, in mol/dm^3^). The partition coefficient (QPlogPo/w) for all compounds, which is essential for predicting absorption in the body, fell within the recommended range (QPlogPo/w = 3.666 to 6.125; limits −2.0 to 6.5) (Table S2, Supporting Information). Among the various properties, the predicted Blood–Brain Barrier (BBB) penetration value (QPlogBB: acceptable range −3.0 to 1.2) was particularly important, as it indicates the molecule’s ability to cross the BBB. The predicted QPlogBB value for compounds **3**, **5**, **6**, **9**, **11**, and **12** (QPlogBB = −0.887 to −1.626; see Table S2, Supporting Information) fell within the optimal penetration range. The Polar Surface Area (PSA) measures the molecule’s hydrogen bonding capacity, and its value should be below a certain threshold for central nervous system activity. Molecules with a PSA <100 Å² are more likely to penetrate the BBB. All compounds had PSA values within this range. They also failed the rule of three [41] once for the QPlogS limit. The compounds also had a high percentage of human oral absorption (78.86 to 96.23%) (Table S2, Supporting Information). Other physicochemical descriptors predicted by QikProp (Table S2, Supporting Information) were found within acceptable limits for human use.

Consequently, this study demonstrates that the designed compounds **3**, **5**, **6**, **9**, **11**, and **12** have suitable pharmacokinetic properties, making them viable candidates for drug development. However, it is important to keep in mind that the presence of the phenyl ring considerably decreases the solubility of the compounds bearing it.

## 3. Conclusions

Sigma receptors (σRs) are appropriate biological targets for the therapy of neuropathic pain. We have recently embarked on a research program targeting the research of new small molecules for the treatment of neuropathic pain [31]. Furthermore, in this work we identified that 2-{[2-(1-benzylpiperidin-4-yl)ethyl]amino}-6-[methyl(prop-2-yn-1-yl)amino]pyridine-3,5-dicarbonitrile (**5**) is an easily available polyfunctionalized small pyridine exhibiting potent dual-target activities against AChE (13 nM), BuChE (3.1 μM), and σ_1/2_R affinity, behaving as a σ_1_R agent, showing *K*_i_ values of 1.45 nM for hσ_1_R and 2.9 nM for gpσ_1_R. As shown in Table 1, compound **5** has around a 1.3-fold higher affinity for *h*σ_1_R than the standards **NE-100** and **PB2**. These results are in good agreement with those found in the molecular modeling of compound **5**. This is possibly due to the preferred combination in this molecule of a linker n = 2 connecting the *N*-Bn-piperidine motif to the C2 pyridine, without a phenyl group at C4, and a *N*-Me-substituted propargyl amine in the chain located at C6.

Based on the promising in silico ADME analysis confirming its potential druggability, work is now in progress in our laboratory to investigate the functional analysis of compound **5** in-depth for its potential use in therapy to treat neuropathic pain.

## 4. Materials and Methods

### 4.1. Biological Assays

#### 4.1.1. In Vitro σ1R Competitive Binding Assay

The *h*σ_1_R competitive binding assay was performed as previously reported [38]. In brief, radioligand competition experiments were performed using preparations from HEK293 cells stably transfected with human σ_1_R cells (provided by Olivier Soriani, Université de Nice Sophia-Antipolis, Nice, France) [42]. The binding of the σ_1_R specific radioligand (+)-[^3^H]pentazocine (1.051 TBq/mmol; PerkinElmer LAS GmbH, Rodgay, Germany) was measured at equilibrium in the presence of the test compounds at different concentrations (10 mM stock solutions in DMSO; final concentrations 10^−5^–10^−11^ M, 0.1 % DMSO) in TRIS buffer at pH 7.4 (50 mM TRIS-HCl, 120 mM NaCl, 5 mM KCl, 2 mM CaCl_2_, 1 mM MgCl_2_). Total binding was measured without the addition of a competitor, and non-specific binding was measured with 10 µM haloperidol as the competitor. The vials were incubated at room temperature (rt) (~21 °C) at 300 rpm. The incubation was terminated after 60 min by vacuum filtration using Whatman^®^ GF/B glass-fiber filtermats (#FPD-100, Brandel Inc., Gaithersburg, MD, USA), soaked with freshly prepared 0.3% polyethyleneimine, using a 48-well harvester (Brandel Inc., Gaithersburg, MD, USA). Filters were washed four times with cold buffer (50 mM TRIS-HCl, pH 7.4 at 4 °C) and placed in scintillation vials. In total, 3 mL of a liquid scintillation cocktail (Ultima Gold TM; PerkinElmer, Waltham, MA, USA) was filled in each vial, and the vials were shaken at rt at 375 rpm for 120 min. Filter-bound radioactivity was measured in a Hidex 600 SL liquid scintillation counter (Hidex, Turku, Finnland). By performing nonlinear regression analysis of the binding curves (GraphPad Prism 3.0, GraphPad Software, Inc., La Jolla, CA, USA), the IC_50_ values were obtained, and the equilibrium inhibition constants *K*_i_ were calculated according to the Cheng–Prusoff equation [36], using the in-house determined equilibrium dissociation constant *K*_D_ of 33 nM (+)-[^3^H]pentazocine. Each test compound was measured in 2–3 independent experiments, each performed in triplicate.

#### 4.1.2. In Vitro σ2R Competitive Binding Assay [38]

Two rat livers were cut into small pieces and homogenized using a potter [Elvehjem Potter (B. Braun Biotech International, Melsungen, Germany); 500–800 rpm, 10 up-and-down strokes] in 6 volumes of cold 0.32 M sucrose. The suspension was centrifuged at 1200× *g* for 10 min at 4 °C. The supernatant was separated and centrifuged at 31,000× *g* for 20 min at 4 °C. The pellet was resuspended in 5–6 volumes of buffer (50 mM TRIS, pH 8.0) and incubated at rt for 30 min. After the incubation, the suspension was centrifuged again at 31,000× *g* for 20 min at 4 °C. The final pellet was resuspended in 5–6 volumes of buffer and stored at −80 °C in 1.5 mL portions containing about 2 mg of protein/mL.

The stock solution of the respective test compound (10 mM in DMSO) was diluted with the assay buffer to obtain the required test solutions for the assay. All binding experiments were carried out in duplicates in 96-well multiplates. The concentrations given are the final concentrations in the assay. The assays were performed with the radioligand [^3^H]-DTG (specific activity 50 Ci/mmol; ARC, St. Louis, MO, USA). The thawed membrane preparation of rat liver (about 100 µg of protein) was incubated with various concentrations of the test compound, 3 nM [^3^H]-DTG, and a buffer containing (+)-pentazocine (500 nM (+)-pentazocine in 50 mM TRIS, pH 8.0) at rt. The non-specific binding was determined with 10 μM non-labeled DTG. The K_d_ value of [^3^H]-DTG is 17.9 nM [43].

Generally, the assays were performed by adding 50 µL of the respective assay buffer and 50 µL of the test compound solution in various concentrations (10^−5^, 10^−6^, 10^−7^, 10^−8^, 10^−9^, and 10^−10^ mol/L), 50 µL of the corresponding radioligand solution, and 50 µL of the respective receptor preparation into each well of the multiplate (total volume 200 µL). The receptor preparation was added last. During the incubation, the multiplates were shaken at a speed of 500−600 rpm at rt. The assays were terminated after 120 min by rapid filtration using the harvester (MicroBeta FilterMate 96; PerkinElmer LAS, Rodgau, Germany) equipped with filtermats presoaked in 0.5% aqueous polyethylenimine solution for 2 h at rt before use. During the filtration, each well was washed five times with 300 µL of water. Subsequently, the filtermats were dried at 95 °C. The solid scintillator (Meltilex; PerkinElmer LAS, Rodgau, Germany) was melted on the dried filtermats at a temperature of 95 °C for 5 min. After solidifying the scintillator at rt, the trapped radioactivity in the filtermats was measured with the scintillation analyzer (MicroBeta Trilux; PerkinElmer LAS, Rodgau, Germany). Each position on the filtermat corresponds to one well of the multiplate and was measured for 5 min with the [^3^H]-counting protocol. The overall counting efficiency was 20%. The IC_50_ values were calculated with the program GraphPad Prism^®^ 3.0 (GraphPad Software, San Diego, CA, USA) by nonlinear regression analysis. Subsequently, the IC_50_ values were transformed into *K*_i_ values using the equation of Cheng and Prusoff [36]. The *K*_i_ values are given as the mean value ± SEM from three independent experiments.

### 4.2. Molecular Simulations

#### Molecular Modeling

Compound **5** as protonated amine was prepared with Discovery Studio (DS), 2022, software package, using standard bond lengths and bond angles. The molecular geometry of the compound was energy-minimized using the adopted-based Newton–Rapson algorithm with the CHARMm force field [44] until the RMS gradient was below 0.01 kcal/mol.Å. The ligand was set up for docking with the help of AutoDockTools (ADT; version 1.5.6), and all the rotatable bonds were allowed to rotate freely. The three-dimensional crystal structure of *h*σ_1_R, which is bound to the antagonist PD144418 (PDB ID: 5HK1; chain B), was obtained from the Protein Data Bank. In the case of σ_2_R, the rat σ_2_R model was retrieved from the SWISS-MODEL Repository [45]. A putative three-dimensional structure of rat σ_2_R was created based on the crystal structure of bovine σ_2_R (PDB ID: 7M93; chain A). Next, the receptor structures were prepared for docking. First, in the PDB crystallographic structure, water molecules, any co-crystallized solvent, and the ligand were removed. Then, proper bonds, bond orders, hybridization, and charges were assigned using the protein model tool in the DS software package. ADT was used to add hydrogen and partial charges using Gasteiger charges and to generate the docking input files. The docking approach included protein flexibility through a set of different conformations of selected side chains into the σ_1_R macromolecule. Using the AutoTors module, to give flexibility to the σ_1_R binding site, side chains of twelve residues lining the site were allowed to move as follows: Tyr103, Glu172, Phe107, Asp126, Val152, Phe146, Gln135, His154, Glu158, Ser117, Tyr120, and Tyr206. The docking box was positioned in the middle of the protein (x = −6.978; y = 20.413; z = −27.539). A grid box of 28 × 22 × 34 with a grid point spacing of 1 Ǻ was used. For σ2R, the grid box was built with a resolution of 1 Å and 40 × 58 × 34 points, and it was positioned in the middle of the protein (x = 9.476; y = 29.642; z = −8.526). AutoDock Vina software (version 1.2.5) [39] was employed for the protein–ligand docking calculations with the default settings except for num_modes, which was set to 40. The more energetically favorable conformation was selected as the best pose. DS software was also used to process the docking results.

## Data Availability

The data presented in this study are included in the article/Supplementary Material. Further inquiries can be directed to the corresponding authors.

## Supplementary Materials

The following supporting information can be downloaded at: https://www.mdpi.com/article/10.3390/ijms26031266/s1.
