# Supplementary material for: 2-{N-[ω-(1-Benzylpiperidin-4-yl)alkyl]amino}-6-[(prop-2-yn-1-yl)amino]pyridine-3,5-dicarbonitriles Showing High Affinity for σ1/2 Receptors
Source: Int J Mol Sci. 2025 Jan 31;26(3):1266. doi: 10.3390/ijms26031266 (PMC11818082; doi:10.3390/ijms26031266)
Supplement: Supplementary file 1 [file ijms-26-01266-s001.zip › ijms-3409207-supplementary.pdf]

Content

Table S1..1
Figure S1...2
Figure S2...3
Figure S3...4
Figure S4...5
Table S2..6

Table S1. IC_{50} values for the inhibition of AChE/BuChE, and MAO A/B by pyridines **1-12.**

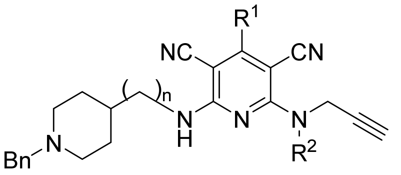

1 R^1, R^2 = H, n= 0
8 R^1 = Ph, R^2 = H, n= 0
11 R^1 = Ph, R^2 = Me, n= 1
2 R^1, R^2 = H, n= 2
5 R^1 = H, R^2 = Me, n= 2
3 R^1, R^2 = H, n= 3
6 R^1 = H, R^2 = Me, n= 3
9 R^1 = Ph, R^2 = H, n= 3
12 R^1 = Ph, R^2 = Me, n= 3
4 R^1, R^2 = H, n= 4
7 R^1 = H, R^2 = Me, n= 4
10 R^1 = Ph, R^2 = H, n= 4

**Compounds**	**AChE[a] (nM)**	**BuChE[b] (nM)**	**MAO A[c] (nM)**	**MAOB[d] (nM)**
1	530 ± 70	n.i.	n.i.	n.i.
**2**	16 ± 2	n.i.	n.i.	n.i.
**3**	4.7 ± 0.5	2030 ± 370	n.i.	n.i.
**4**	1.3 ± 0.3	530 ± 60	n.i.	n.i.
**5**	**13 ± 1**	**3100 ± 300**	**n.i.**	**n.i.**
**6**	6.2 ± 1.4	1120 ± 160	6110 ± 1400	n.i.
**7**	1.1 ± 0.3	600 ± 80	3950 ± 940	n.i.
**8**	4000 ± 100	n.i.	25 ± 1	n.i.
**9**	19 ± 5	n.i.	n.i.	n.i.
**10**	1.7 ± 0.3	840 ± 100	n.i.	n.i.
**11**	270 ± 52	5000 ± 700	n.i.	n.i.
**12**	14 ± 1	230 ± 30	n.i.	n.i.

[a] EeAChE; [b] eqBuChE; [c] human MAO A; [d] human MAO B ; n.i.: no inhibition.

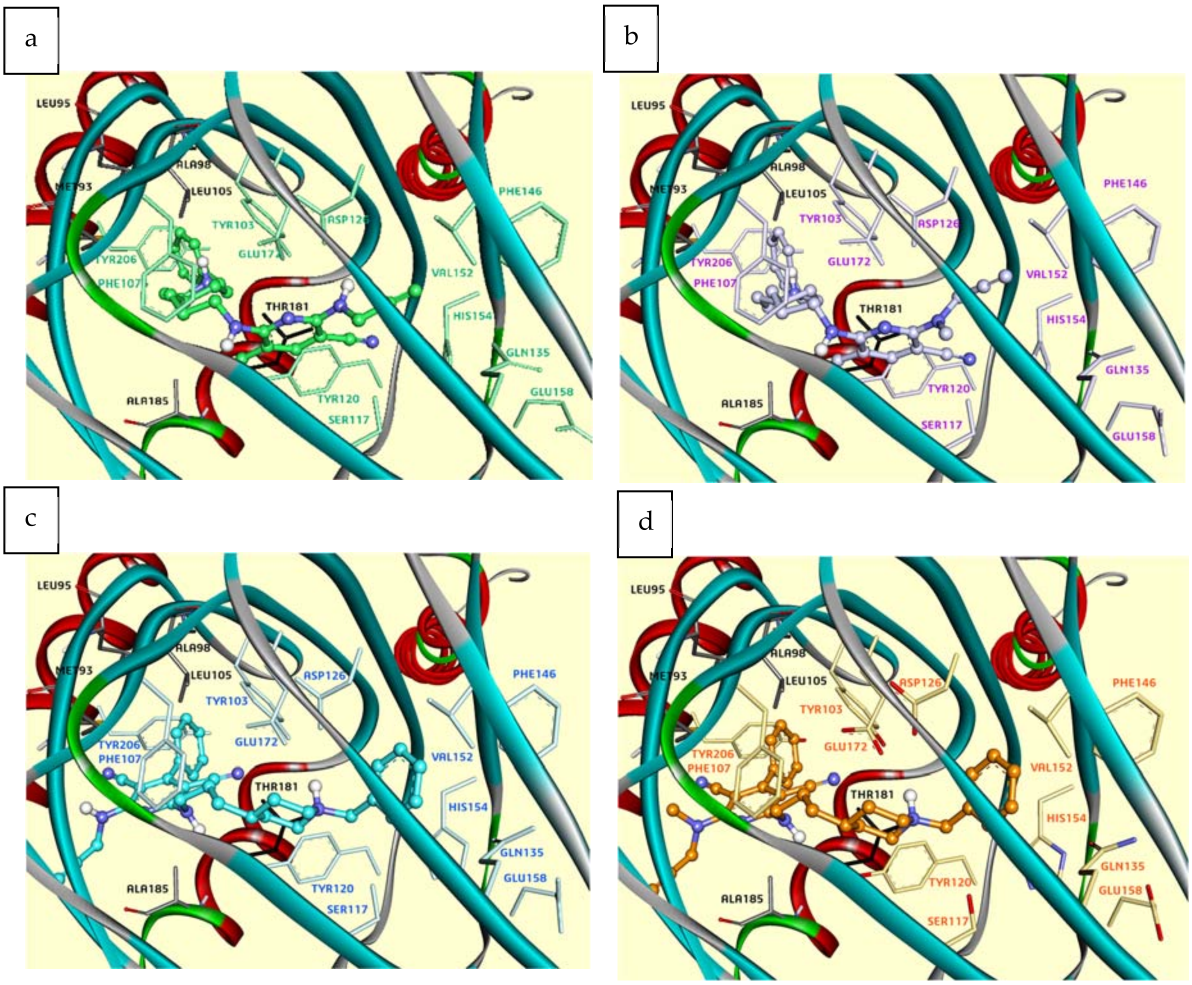

Figure S1. Docking poses of compounds **3** (green color balls and sticks), **6** (violet color balls and sticks), **9** (blue color balls and sticks), and **12** (orange color balls and sticks) in the active site σ1R. Flexible residues are shown in the same color as the ligands.

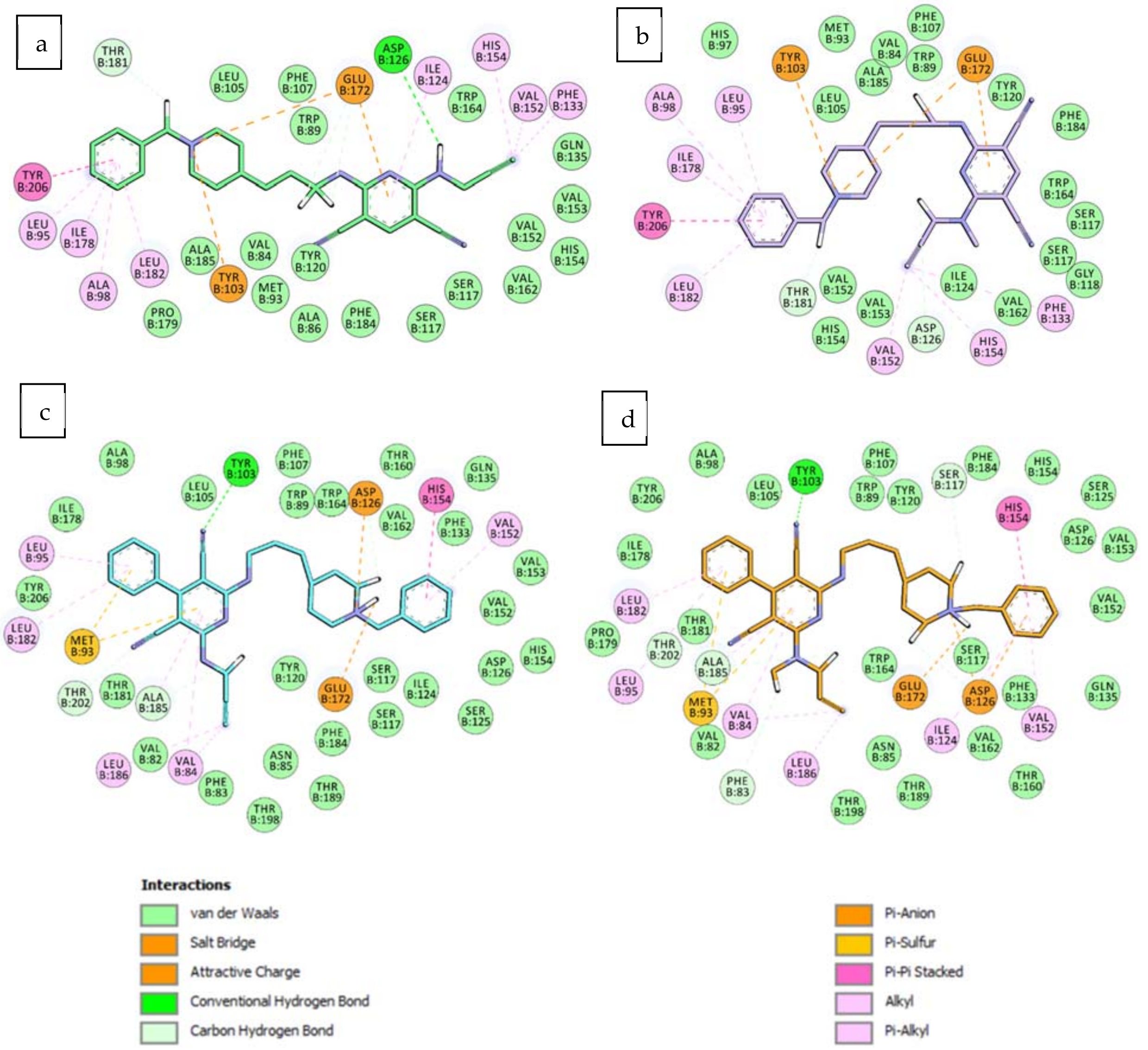

Figure S2. Ligand-receptor interactions of compounds **3** (green color stick), **6** (violet color stick), **9** (blue color stick) and **12** (orange color stick) in the active site of σ1R.

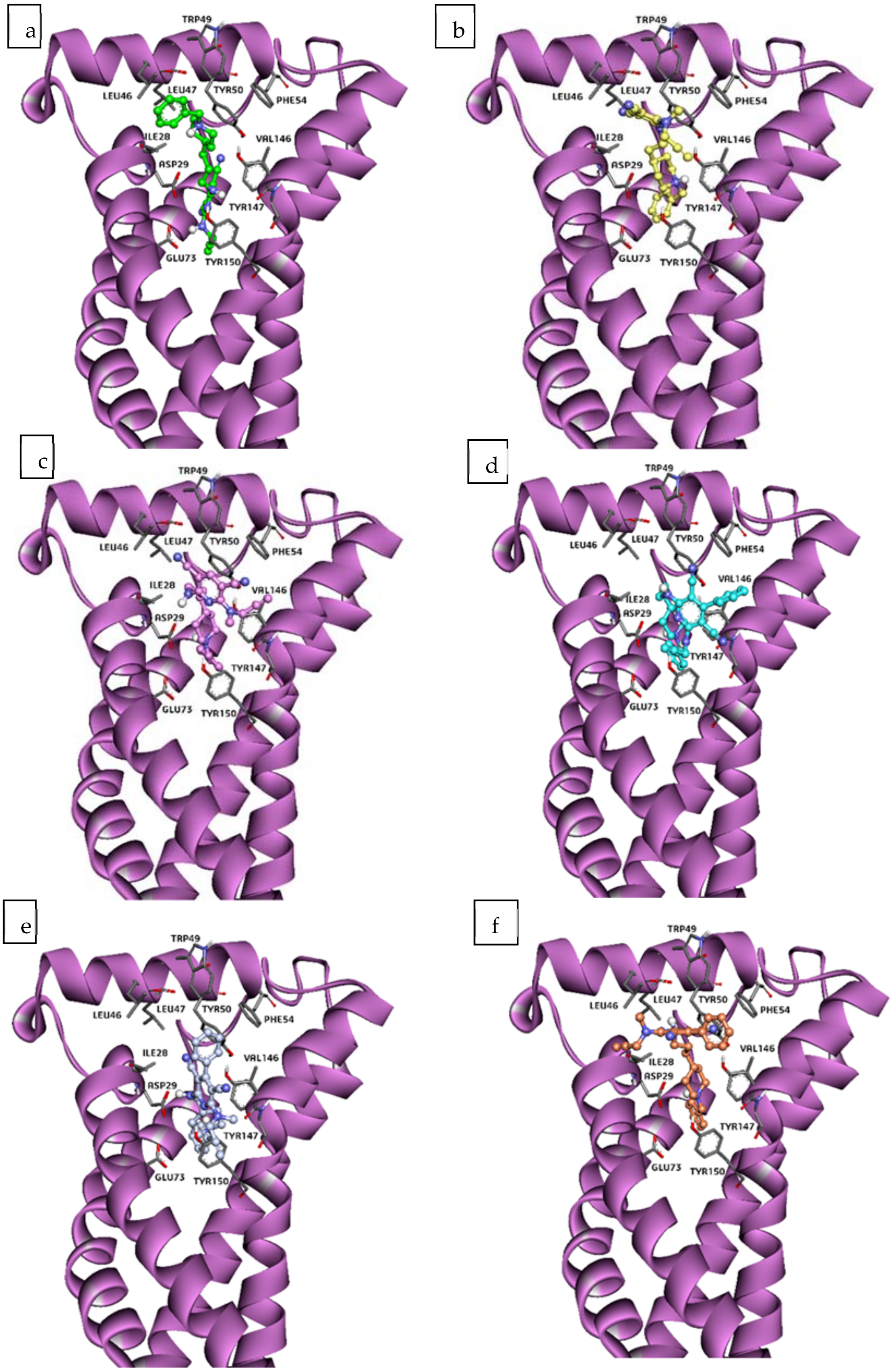

Figure S3. Docking poses of compounds **3** (green color), **5** (yellow color), **6** (pink color), **9** (blue color), **11** (violet color) and **12** (orange color) in the active site of modeled rat σ2R.

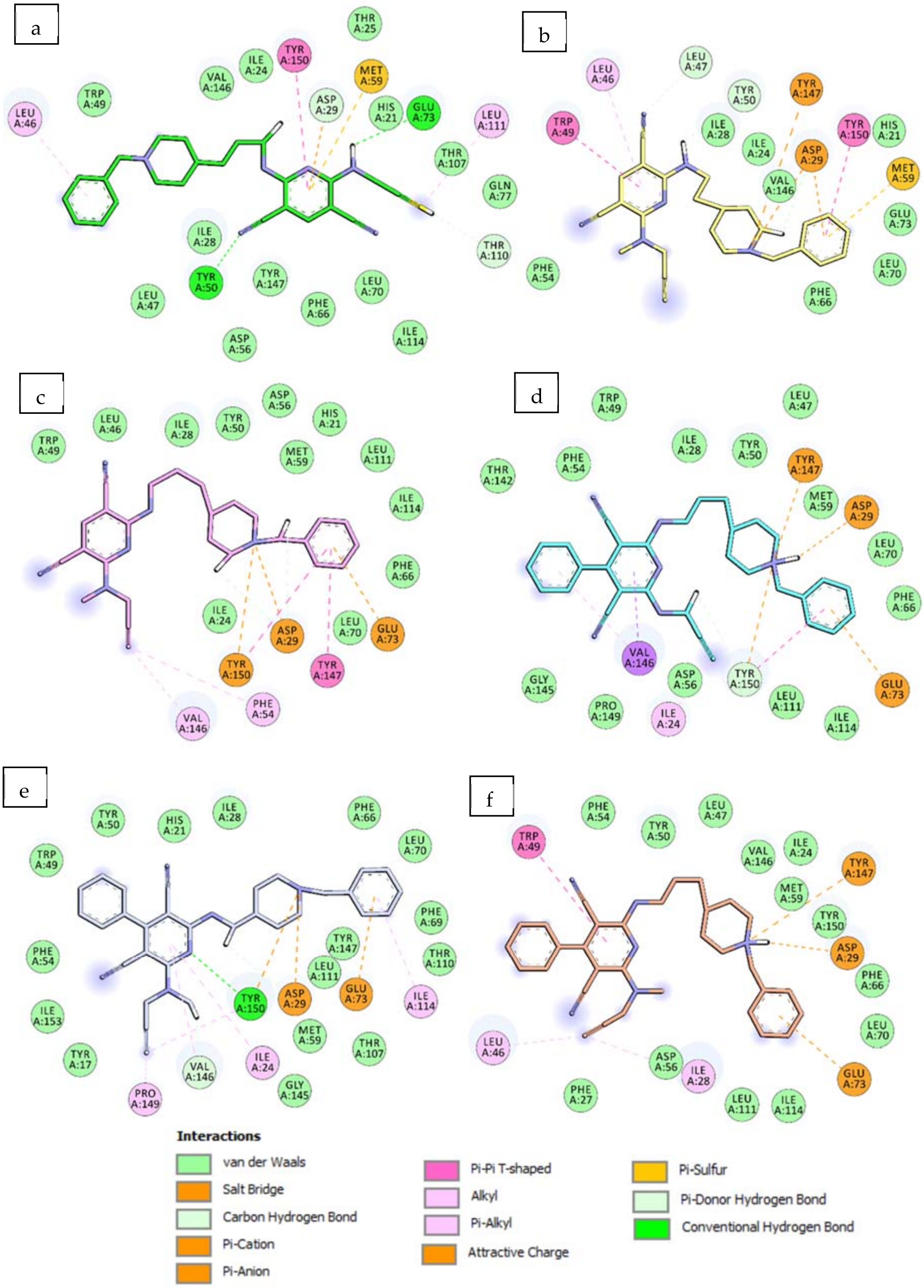

Figure S4. Ligand-receptor interactions of compounds **3** (green color stick), **5** (yellow color stick), **6** (pink color stick), **9** (blue color stick), **11** (violet color stick) and **12** (orange color stick) in the active site of modelled rat σ2R.

Table S2. Physicochemical properties for compounds **3**, **6**, **9**, **11** and **12** calculated using QikProp.

Compound	MW	SASA	volume	donorHB	accptHB	QPlogPo/w	QPlogS	QPPCaco
**3**	412.536	798.400	1414.131	2.500	7.000	3.666	-5.437	70.446
**5**	412.536	799.155	1421.404	1.500	7.000	3.996	-6.156	102.577
**6**	426.563	831.835	1483.068	1.500	7.000	4.449	-5.776	126.528
**9**	488.634	904.336	1639.414	2.500	7.000	5.420	-7.310	127.331
**11**	474.607	781.632	1500.876	1.500	7.000	4.855	-7.106	191.733
**12**	502.661	929.952	1697.637	1.500	7.000	6.125	-7.677	221.120

Compound	PSA	QPlogBB	metab	QPlogKhsa	% HOA	ROF	ROT
**3**	89.556	-1.626	3	0.480	81.485	0	1
**5**	80.559	-1.361	3	0.593	86.336	0	1
**6**	79.283	-1.348	3	0.711	90.618	0	1
**9**	89.328	-1.459	3	1.022	83.395	1	1
**11**	72.676	-0.887	3	0.834	96.226	0	1
**12**	78.602	-1.167	3	1.244	78.857	2	1

MW: Molecular weight of the molecule (130.0-725.0). SASA: Total Solvent Accessible Surface Area, in square angstroms, using a probe with a 1.4Å radius (limits 300.0-1000.0). volume: Total solvent-accessible volume, in cubic angstroms, using a probe with a 1.4 Å radius (limits 500.0-2000.0). donorHB: Estimated number of hydrogen bonds that would be accepted by the solute (limits: 2.0-20.0). accptHB: Estimated number of hydrogen bonds that would be donated by the solute (limits: 0.0-6.0). QPlogPo/w: Predicted octanol/water partition coefficient (limits -2.0-6.5). QPlogS: Predicted aqueous solubility. S, in mol/dm^3, is the concentration of the solute's saturated solution that is in equilibrium with crystalline solid (limits -6.5-0.5). QPPCaco: Predicted apparent Caco-2 cell permeability in nm/sec. Caco-2 cells is a model for the gut-blood barrier. QikProp predictions are for non-active transport. (< 25 poor, > 500 great). PSA: Van der Waals surface area of polar nitrogen and oxygen atoms (limits 7.0-200.0). QPlog BB: Predicted brain/blood partition coefficient (limits -3.0–1.2). metab: Number of likely metabolic reactions (limits 1-8). QPlogKhsa: Prediction of binding to human serum albumin (limits -1.5-1.5). HOA: Predicted qualitative Human Oral Absorption on 0 to 100% scale. ROF: Number of violations of Lipinski's Rule of Five (molecular weight < 500, QPlogPo/w < 5, number of hydrogen bond donor ≤ 5, number of hydrogen bond acceptors HB ≤ 10). ROT: Number of violations of Jorgensen's rule of three (QPlogS> -5.7, QPCaco> 22 nm/s, number of primary metabolites < 7).
